# A novel mutation in the *WFS1 *gene identified in a Taiwanese family with low-frequency hearing impairment

**DOI:** 10.1186/1471-2350-8-26

**Published:** 2007-05-22

**Authors:** Hsun-Tien Tsai, Ying-Piao Wang, Shing-Fang Chung, Hung-Ching Lin, Guan-Min Ho, Min-Tsan Shu

**Affiliations:** 1 Department of Otolaryngology, Mackay Memorial Hospital, 92, Section 2, Chungshan North Road, Taipei, Taiwan; 2 Department of Medical Research, Mackay Memorial Hospital, 45, Minsheng Road, Tamshui, Taipei, Taiwan; 3 Department of Speech and Hearing Disorders and Sciences, National Taipei College of Nursing, 89 Nei-Chiang Street, Wanhua, Taipei, Taiwan

## Abstract

**Background:**

Wolfram syndrome gene 1 (*WFS1*) accounts for most of the familial nonsyndromic low-frequency sensorineural hearing loss (LFSNHL) which is characterized by sensorineural hearing losses equal to and below 2000 Hz. The current study aimed to contribute to our understanding of the molecular basis of LFSNHL in an affected Taiwanese family.

**Methods:**

The Taiwanese family with LFSNHL was phenotypically characterized using audiologic examination and pedigree analysis. Genetic characterization was performed by direct sequencing of *WFS1 *and mutation analysis.

**Results:**

Pure tone audiometry confirmed that the family members affected with LFSNHL had a bilateral sensorineural hearing loss equal to or below 2000 Hz. The hearing loss threshold of the affected members showed no progression, a characteristic that was consistent with a mutation in the *WFS1 *gene located in the DFNA6/14/38 locus. Pedigree analysis showed a hereditarily autosomal dominant pattern characterized by a full penetrance. Among several polymorphisms, a missense mutation Y669H (2005T>C) in exon 8 of *WFS1 *was identified in members of a Taiwanese family diagnosed with LFSNHL but not in any of the control subjects.

**Conclusion:**

We discovered a novel heterozygous missense mutation in exon 8 of *WFS1 *(i.e., Y669H) which is likely responsible for the LFSNHL phenotype in this particular Taiwanese family.

## Background

Severe hearing impairment affects 1 in 1000 newborns and will in a significant percentage of this population lead to a delayed speech and language development. Hereditary hearing loss may be associated with other clinical disorders and coexist with other organ malformations (syndromic), or may be the only disease phenotype (non-syndromic). Approximately 70% of hereditary deafness is non-syndromic, of which ~80% is categorized as autosomal recessive (DFNB), ~15–20% as autosomal dominant (DFNA), ~1% as X-linked (DFN), and ~≥1% as mitochondrial or chromosome aberrations [1]. To date, over 90 different loci correlated with the non-syndromic disease have been mapped [2]. Thirty-nine genes underlying non-syndromic hearing impairment have been identified and more than a dozen genes involved in syndromic deafness have also been uncovered [3].

Autosomal dominant non-syndromic low frequency sensorineural hearing loss (LFSNHL; OMIM #600965) is a unique phenotype of hearing impairment affecting the low frequencies (i.e., 2000 Hz and below) [4]. Although over 50 loci have been mapped in various types of DFNA cases, to date, only three chromosomal loci have been associated with LFSNHL (i.e., 5q31 (DFNA1), 4p16 (DFNA6/14/38), and 5q31 between D5S1972 and D5S410 (DFNA54)) [2,5]. Two genes, namely the *Homo sapiens *diaphanous homolog 1 gene (*DIAPH1*) related to DFNA1 and the Wolfram syndrome 1 gene (*WFS1*), have been shown to be involved in LFSNHL [6,7]. *WSF1 *encodes wolframin, a hydrophobic transmembrane protein that is related to LFSNHL associated with DFNA6/14/38 [8,9]. Although the loci DFNA1, DFNA6/14/38, and DFNA54 are associated with LFSNHL, the progression of hearing impairment caused by a mutation in either of these three loci exhibits different characteristics. Patients carrying a mutation in the DFNA1 locus typically experience a rapidly deteriorating hearing impairment spreading from low to all higher frequencies [10]. In contrast, patients carrying a mutation in the DFNA54 or DFNA6/14/38 locus experience deafness propagating from low to high frequencies [5] or have no or mild progression beyond the presbycusis [4,9,11], respectively.

In this study, we have examined a Taiwanese family affected by non-progressive LFSNHL and identified a novel mutation in the *WFS1 *gene (i.e., Y669H) which is most likely correlated with LFSNHL as this mutation was not observed in any of the numerous control subjects with normal hearing abilities who were examined herein.

## Methods

### Subjects and audiometric examination

A Taiwanese family comprised of 10 individuals, including three members diagnosed with LFSNHL, was examined. After signing the informed consent, family members received a medical survey, including otorhinolaryngologic examination, especially otoscopy. Based on the medical history of the subjects tested herein, syndromic and/or environmental deafness could be excluded based on the review of the medical history (data not shown). Unrelated control subjects exhibited no symptomatic signs of auditory and/or vestibular impairment, based on their medical history and physical examination, including pure tone audiometry (GSI 17 portable audiometer, (Grason-Stadler, Inc., Madison, WI, USA).

### DNA extraction

Genomic DNA was extracted from 4 ml whole blood or buccal epithelial cells using the GFX™ Genomic Blood DNA Purification Kit (Amersham Biosciences) and stored at -20°C until used.

### Primer design and PCR amplification

Coding and flanking intron sequences of exons 2 to 8 of the *WFS1 *gene (GenBank accession number AC004599) were amplified by PCR using 13 primer pairs (Table 1). PCR reactions were performed in a 50-μL volume containing 100–500 ng DNA, 0.2 μM of each forward and reverse primer, 200 μM dNTPs, 1× PCR buffer (10 mM Tris-HCl pH 8.3, 50 mM KCl, 2.5 mM MgCl_2_), and 0.5 U of Ampli*Taq *Gold™ DNA Polymerase (Applied Biosystems, Foster City, CA, USA). PCR amplifications were performed in a Gene Amp PCR System 9700 (Applied Biosystems) with an initial denaturation step at 95°C for 10 min and then 36 cycles at 95°C for 30 s, 60°C for 30 s, 72°C for 45 s, followed by 7 min of final extension at 72°C. In case of exon 8, amplifications were performed for 30 cycles at an annealing temperature of 58°C.

**Table 1:** Primers used in PCR/sequencing reaction of *WFS1 *coding region*

Primer sequence		
Fragment	Forward primer (5-3)	Reverse primer (5-3)

Exon 2^†^	AAGCGGTGCTGGCCCATG	CCGTTCCCACCCAGCTATC
Exon 3^†^	TACTCCTGGCCTGGATTTGA	CATGGGCACCCTACCAACA
Exon 4^†^	AGTGGCCGGAGGCTCAGT	CCAACAGCATCACCAGCGT
Exon 5^†^	AGTCAGATGTCCATGCATCC	CTCTACAGGAAGGTTCTGGT
Exon 6^†^	GAGCACGCTACGTGGTGCT	GGAGGCACGGGTGAGATAG
Exon 7^†^	CCTGAACCCACTCAGCTC	CCAGCGGCACGGCTGTAA
Exon 8-1^§^	TTCCCACGTACCATCTTTCC	CACATCCAGGTTGGGCTC
Exon 8-2^§^	AGAACTTCCGCACCCTCAC	TCAGGTAGGGCCAATTCAAG
Exon 8-3^§^	CTATCGCTGCTGCCCTCC	GGGCAAAGAGGAAGAGGAAG
Exon 8-4^§^	GTGAGCTCTCCGTGGTCATC	CCCTCTGAGCGGTACACATAG
Exon 8-5^§^	ATCCTGGTGTGGCTCACG	GTAGAGGCAGCGCATCCAG
Exon 8-6^§^	GCGTGACTGACATCGACAAC	GCTGAACTCGATGAGGCTG
Exon 8-7^§^	CAGCAGCGAGTTCAAGAGC	CCTCATGGCAACATGCAC

*WFS1 sequence based on the GenBank entry (accession number: AC004599).

^†^Primers were designed and synthesized herein (Methods).

^§^Primers were provided by Dr. Yann-Jinn Lee.

### Direct sequencing

A 10-μL sequencing reaction was performed with 100 ng of the purified PCR product as a template, 30 pmol of each of the sequencing primers, and 1 μL of the BigDye Terminator v3.0 Sequencing Ready Reaction Cycle sequencing kit (Applied Biosystems). After completion of the reaction, the mixture was purified by gel filtration on a Sephadex G-50 column (Amersham Pharmacia Biotech, Piscataway, NJ, USA). Bidirectional sequencing was analyzed on an automated ABI Prism 377-18 DNA sequencer (Applied Biosystems).

### PCR-RFLP analysis

A PCR-based restriction fragment length polymorphism (RFLP) assay was used to detect the 1235 T>C and 2005 T>C nucleotide changes. The 672-bp PCR product amplified by PCR using the forward primer (5'-TTC CCA CGT ACC ATC TTT CC-3') and the reverse primer (5'-CAC ATC CAG GTT GGG CTC-3') contained a polymorphic restriction site at position 1235. Amplification was performed as described above and PCR products were analyzed by 2% agarose gel electrophoresis. PCR products (3 μL) were digested with 7 U of restriction enzyme *Bbs*I (New England Biolabs, Ipswich, MA, USA) in a final volume of 10 μL and fragments were separated on a 2% agarose gel.

The 154-bp PCR product with the polymorphic restriction site at position 2005 was amplified by PCR using forward primer 5'-GTC AAG CTC ATC CTG GTG TG-3' and reverse primer 5'-CCA TGT TGG TCT CCT TCC AG-3'. PCR amplification was performed as described above. After verification of the PCR products on agarose gel, 3 μL was used to digest with 7.5 U of *Nla*III (New England Biolabs) in a final volume of 10 μL and fragments were separated on a 4% agarose gel.

## Results

### Characteristic hearing impairment in family members affected with LFSNHL

**Figure 1:**
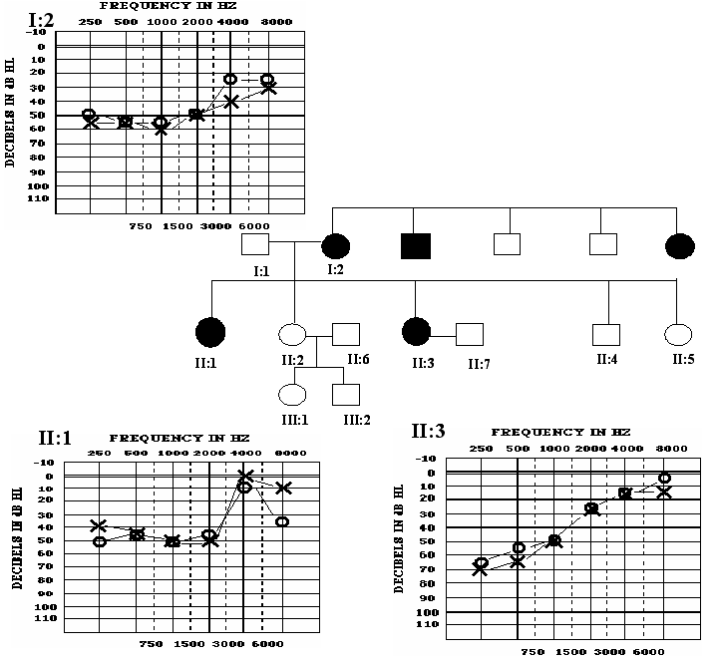
Pedigree of the family affected with LFSNHL and audiogram of three chosen affected individuals. In the pedigree, shaded symbols indicate family members affected by LFSNHL, while open symbols indicate those with a normal hearing. An autosomal dominant hereditary pattern is shown in this pedigree. Pure tone audiograms of I:2, II:1, and II:3 show a predominantly bilateral low-tone hearing impairment. Circles and crosses indicate air conduction in the right and left ears respectively.

The Taiwanese family examined herein showed an autosomal dominant hereditary pattern with a full penetrance, which strongly suggests that an underlying genetic factor is involved (Figure 1). Three affected members (I:2, II:1, and II:3) had a characteristic LFSNHL and did not wear hearing aids. Audiograms of the affected members showed a bilateral average hearing of 40–60 dB at the frequencies of 0.25, 0.5, 1, and 2 kHz and a normal hearing threshold at 4 and 8 kHz (Figure 1).

The hearing loss threshold of the mother (I:2, age 56) and her affected daughters (II:1, age 36; and II:3, age 22) showed nearly no progression based on a comparison of symptoms between the mother and her daughters. The latter is characteristic for a mutation in the DFNA6/14/38 locus of *WFS1 *(Figure 1).

### Identification of a novel mutation in the *WFS1 *gene in family members with LFSNHL

Four non-synonymous nucleotide changes were identified by direct sequencing of the *WFS1 *gene coding sequence (exon 2–8) in the three affected individuals. Two of these changes (1832A>G and 2158A>G) were ruled out by phenotype-genotype comparisons within the family, whereas the other two non-synonymous changes (1235T>C and 2005A>C) were potential candidate mutations.

**Figure 2:**
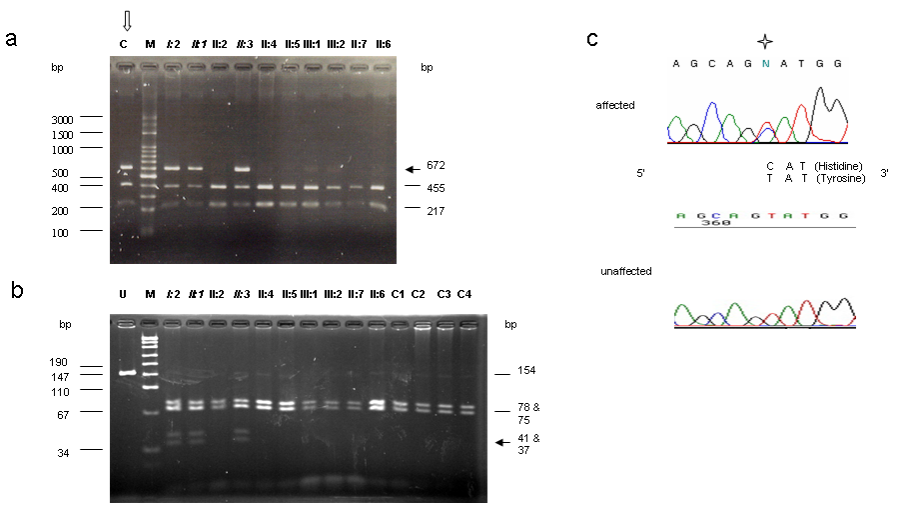
**(a) **RFLP of the *Bbs*I-digested PCR fragments flanking the 1235T>C region for the family affected by LFSNHL and one normal control. The 672-bp PCR product obtained using the forward primer (5'-TTC CCA CGT ACC ATC TTT CC-3') and the reverse primer (5'-CAC ATC CAG GTT GGG CTC-3') contains a polymorphic restriction site at position 1235. Two fragments of 455 bp and 217 bp from genotype TT were unaffected by enzyme digestion, while three fragments of 672 bp (solid arrow), 455 bp, and 217 bp were observed from heterozygotes carrying genotype TC as I:2, II:1, II:3 and one normal control (C indicated by the open arrow). M: DNA marker. **(b) **RFLP of *Nla*III digested the fragment flanking the 2005T>C region of LFSNHL family members and normal controls. The 154-bp PCR product of the polymorphic restriction site at position 2005 was amplified by PCR using the forward primer (5'-GTC AAG CTC ATC CTG GTG TG-3') and reverse primer (5'-CCA TGT TGG TCT CCT TCC AG-3'). Genotype TT such as unaffected individuals and controls (C1, C2, C3 and C4) demonstrated two fragments of 78 and 75 bp, whereas the heterozygote TC such as I:2, II:1, and II:3 showed 4 fragments of 78, 75, 41 (solid arrow), and 37 bp (solid arrow). In all samples, the enzyme also generated a fragment of 1 bp, which was too small to be seen in the gel. U: undigested control, M: DNA marker. **(c) **Electrophoregrams of *WFS1 *gene sequences flanking position 2005 of the affected and unaffected family members. The affected family members are heterozygote (T and C) in position 2005 (labeled with a star), while the unaffected family members are homozygous as T in the same place.

Though 1235T>C had been reported as polymorphism based on study of Japanese society, we compared the RFLP pattern of the family members with that of 100 unrelated controls possessed of Taiwanese genetic background and normal pure tone audiograms [12]. The controls were screened for the occurrence of mutations of interest based on the RFLP assays. The three family members affected with LFSNHL and 1 out of 100 controls (200 chromosomes) had the same RFLP pattern after digestion of the amplicons by *Bbs*I in position 1235 (Figure 2a). The presence of this polymorphism was attributed to a 1235T>C mutation. However, the RFLP pattern of none of the 100 controls (200 chromosomes) was identical to that of any of the affected family members upon digestion of amplicons with *Nla*III at position 2005 (Figure 2b), strongly suggesting that the 2005T>C, but not the 1235T>C mutation, is related to the symptoms observed among the family members.

**Figure 3:**
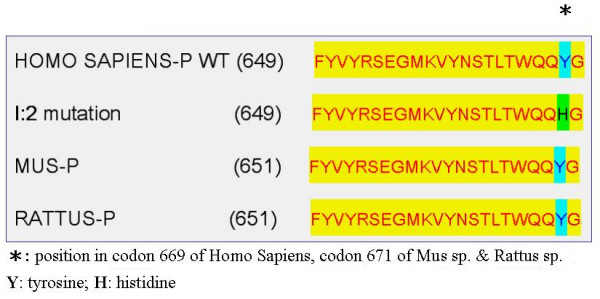
Amino acid sequence comparison of the respective genetic regions of *WFS1 *exon 8 of healthy *Homo sapiens*, *Mus *sp., and *Rattus *sp. indicating its conservativeness.

This demonstrates that we have identified a novel heterozygous mutation 2005T>C in exon 8, which predicts a Y669H alteration (Figure 3). This mutation was found in all the affected members of this family that exhibited LFSNHL (Figure 2c), but not in any of the control subjects, nor was it present in the respective *WFS1 *gene products of *Mus *sp. and *Rattus *sp. (Figure 3). This particular genetic region of the *WFS1 *gene product is highly conserved among healthy humans, *Mus *sp., and *Rattus *sp. (Figure 3).

## Discussion

We have identified a novel mutation in the *WFS1 *gene (i.e., Y669H) in a Taiwanese family whose members were likely diagnosed with LFSNHL, although other diagnoses could not be excluded with certainty. Homozygous or compound heterozygous mutations in *WFS1 *may lead to the autosomal recessive inherited Wolfram syndrome, presenting a variety of phenotypes, including diabetes insipidus, diabetes mellitus, optic atrophy, and deafness, referred to as DIDMOAD (OMIM:#222300) [13]. Audiograms of patients diagnosed with Wolfram syndrome typically show hearing impairment involving the middle and high frequencies [14]. In contrast, heterozygous mutations in *WFS1 *clustered in the C-terminal domain affect the low frequency component of human audiogram [15]. These characteristics are consistent with those presented by the affected subjects of the Taiwanese family studied herein, i.e. low pitch deterioration and the occurrence of a Y669H mutation in the C-terminal region of *WFS1*. Interestingly, a Y669C mutation has been observed in a Wolfram syndrome patient [13]. Therefore, it is likely that the C-terminal domain of *WFS1 *be involved in tonotopic arrangement. However, the role of wolframin and its implication in the etiology of LFSNHL in humans is still unclear. It has been suggested that wolframin could be involved in proper ion transport in the inner ear [16].

The identification of the Y669H mutation in the *WFS1 *gene product of members of the Taiwanese family with symptoms of LFSNHL, the absence of this mutation in control subjects with a normal hearing ability, and the degree of conservation of Y669 in the WFS1 protein of human, mouse, and rat supports our interpretation that the Y669H mutation is causative. A recent examination of a Danish family revealed a clinical spectrum with considerable overlap between classical Wolfram syndrome and non-syndromic LFSNHL [17]. Unfortunately, due to a limited clinical history of the Taiwanese family members lacking endocrinological, ophthalmological, and psychiatric analyses, it is at present impossible to verify whether the symptoms of the subjects studied herein resemble those related to this clinical spectrum. In addition, one should consider that a mutation in one gene may produce multiple phenotypes (allelism) and that a mutation in the same allele may produce different hearing loss characteristics in different families (pleiotropic effect). Furthermore, a recent report strongly suggests that the phenotypic outcome of a particular mutation in a genetic region that causes an improper functioning of the hearing apparatus may be influenced by modifier genes that affect for instance disease penetrance and progression. In other words, the same mutation may lead to a considerably different phenotype in two distinct families as each individual is born with a unique complement of modifier genes [18]. Thus, in order to link with certainty the Y669H mutation in *WFS1 *with symptoms of LFSNHL, future research will involve the construction of a transgenic mouse model carrying this mutation and the analysis of its phenotypic behavior, particularly in respect to symptoms of hearing impairment.

## Conclusion

Phenotypic and genetic characterization of a Taiwanese family affected with LFSNHL revealed a novel missense mutation in *WFS1 *(i.e., 2005A>C; Y669H) which was not observed in any of the examined healthy control subjects. The molecular diagnosis of this family may provide new insights for genetic counseling of families affected with hearing loss and may help to solve the molecular basis of hearing impairment. Evaluation of other affected family members and other unrelated LFSNHL families may help to uncover new *WFS1 *mutations and polymorphisms and help to decipher the role of wolframin in auditory functions.

## Abbreviations

LFSNHL, low-frequency sensorineural hearing loss; RFLP, restriction fragment length polymorphism; WFS1, Wolfram syndrome gene 1.

## Competing interests

The author(s) declare that they have no competing interests.

## Authors' contributions

H.T. Tsai elaborated the design of the study and performed medical genetics analysis. Y. P. Wang was involved in sample collection. S. F. Chung carried out the molecular genetic studies. H. C. Lin performed the audiologic characterizations. G. M. Ho provided critical feedback on the manuscript. M.T. Shu helped with the statistical analyses. All authors read and approved the final manuscript.

## Pre-publication history

The pre-publication history for this paper can be accessed here:


